# Trends in Antifungal Resistance Among *Candida* Species: An Eight-Year Retrospective Study in the Galveston–Houston Gulf Coast Region

**DOI:** 10.3390/jof11030232

**Published:** 2025-03-19

**Authors:** Michael D. Nguyen, Ping Ren

**Affiliations:** Department of Pathology, University of Texas Medical Branch, Galveston, TX 77555, USA; minguye5@utmb.edu

**Keywords:** *Candida* species, antifungal resistance, minimal inhibitory concentration, epidemiological cutoff value, echinocandins, fluconazole

## Abstract

Fungal systemic infections are a growing global health concern, particularly among immunocompromised individuals. *Candida* species are the leading cause of invasive fungal infections, with *C. albicans* historically being the most prevalent. The emergence of *C. auris*, known for its multidrug resistance, presents additional challenges for treatment and infection control. This study retrospectively analyzed minimal inhibitory concentration (MIC) data for common *Candida* species isolated from patients in the Galveston–Houston Gulf Coast region from the EPIC Laboratory Information System (LIS) between October 2016 and September 2024. Antifungal susceptibility was assessed using the Sensititre^TM^ YeastOne^TM^ YO9 AST Plate and interpreted per Clinical and Laboratory Standard Institute (CLSI) guidelines. A total of 1206 clinical yeast isolates from over 29 species were identified, with *Candida* species accounting for 94.5% (1140). *C. albicans* (30.7%), *C. glabrata* (23.5%), *C. parapsilosis* (12.2%), and *C. tropicalis* (10.4%) were the most prevalent. *C. auris* (6.2%) emerged in late 2021 in our region, showing high MICs against fluconazole (92%) and amphotericin B (32.2%). While *C. albicans, C. parapsilosis*, and *C. tropicalis* remained susceptible to echinocandins, fluconazole resistance showed an increasing trend. *C. glabrata* exhibited variable susceptibility to both echinocandins and azoles. These findings highlight the urgent need for enhanced antifungal stewardship, improved diagnostics, and novel therapeutic strategies. Continued regional surveillance and targeted interventions are essential to mitigating the impact of antifungal resistance.

## 1. Introduction

Invasive fungal infections have become a significant challenge in healthcare, particularly among hospitalized and immunocompromised patients [1]. *Candida* species are among the leading causes of bloodstream infections (candidemia), endocarditis, and other systemic complications [2,3]. While *C. albicans* has historically been the most prevalent species [4,5], the incidence of non-*albicans Candida* species, such as *C. glabrata*, *C. parapsilosis*, and *C. tropicalis* has risen, often exhibiting reduced susceptibility to antifungal agents [6,7].

The emergence of *C. auris* has further intensified these concerns regarding antifungal resistance [8]. First identified in 2009, *C. auris* is particularly worrisome due to its multidrug resistance, high transmissibility in healthcare settings, and environmental persistence [9,10]. Global outbreaks have been reported, with some isolates displaying resistance to all three major antifungal classes—azoles, echinocandins, and polyenes—leading to limited treatment options and increased risk of therapeutic failures [11].

Echinocandins remain the first-line treatment for candidemia and other invasive *Candida* infections due to their fungicidal activity and favorable safety profile [12]. While most *C. albicans*, *C. parapsilosis*, and *C. tropicalis* isolates remain susceptible, echinocandin resistance has been increasingly reported in *C. glabrata* and *C. auris*, particularly in patients with prior echinocandin exposure. Resistance in *C. glabrata* is primarily driven by *FKS1* and *FKS2* mutations, which alter drug targets and reduce susceptibility [13,14]. Similarly, *C. auris* isolates with *FKS1* mutations have demonstrated reduced echinocandin susceptibility, raising concerns about treatment limitations [15].

Given these challenges, continuous surveillance of antifungal resistance patterns is crucial for guiding clinical decision-making, optimizing treatment strategies, and strengthening antifungal stewardship programs. Understanding local resistance trends can also help inform infection control measures to curb the spread of resistant strains. This study analyzed antifungal resistance trends among common *Candida* species isolated from our patient population in the Galveston–Houston Gulf Coast area over an eight-year period, with a particular focus on the emergence of *C. auris*.

## 2. Materials and Methods

This retrospective study examined antifungal susceptibility data from clinical *Candida* isolates collected between October 2016 and September 2024 in the Galveston–Houston Gulf Coast region. Isolates were obtained from blood, tissues, and sterile body fluids, etc. Minimum inhibitory concentrations (MICs) for fluconazole, voriconazole, anidulafungin, caspofungin, and micafungin were retrieved from the EPIC Laboratory Information System (LIS). Antifungal susceptibility testing was performed using the Sensititre^TM^ YeastOne^TM^ YO9 AST Plate (ThermoFisher Scientific, Waltham, MA, USA), with resistance breakpoints applied according to the Clinical and Laboratory Standards Institute (CLSI) guidelines [16,17,18]. In instances where no breakpoints have been established, epidemiological cutoff value (ECV) were used to find upper limit of the wild type (UL-WT) values [19], which help to count non-wild-type isolates. Resistance rates were calculated, stratified by species and antifungal class, and analyzed for temporal trends using Excel for Microsoft 365 (https://www.microsoft.com accessed on 25 February 2025) and RStudio Version 2024.04.1+748 (https://www.rstudio.com accessed on 16 March 2025). Statistical analysis included linear regression models to evaluate resistance trends over time. The t-test was used to determine the *p*-value, and <0.05 considered statistically significant.

## 3. Results

A total of 1206 clinical yeast isolates representing over 29 species were included in this study (Supplement Table S1). *Candida* species accounted for 94.5% (1140) of the isolates, with *C. albicans* (370, 30.7%), *C. glabrata* (283, 23.5%), *C. parapsilosis* (147, 12.2%), and *C. tropicalis* (125, 10.4%) being the most prevalent. *C. auris* (75 isolates, 6.2%) was identified starting from the fourth quarter of 2021 in our region.

**Figure 1:**
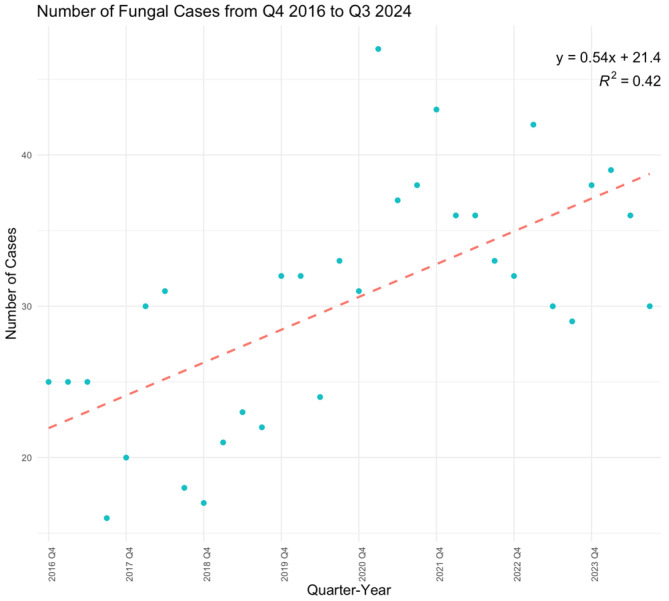
A linear regression model showed a significant increase in fungal cases from quarter 4 (Q4) of 2016 to quarter 3 (Q3) of 2024 (*p* < 0.0001). Q: quarter.

**Figure 2:**
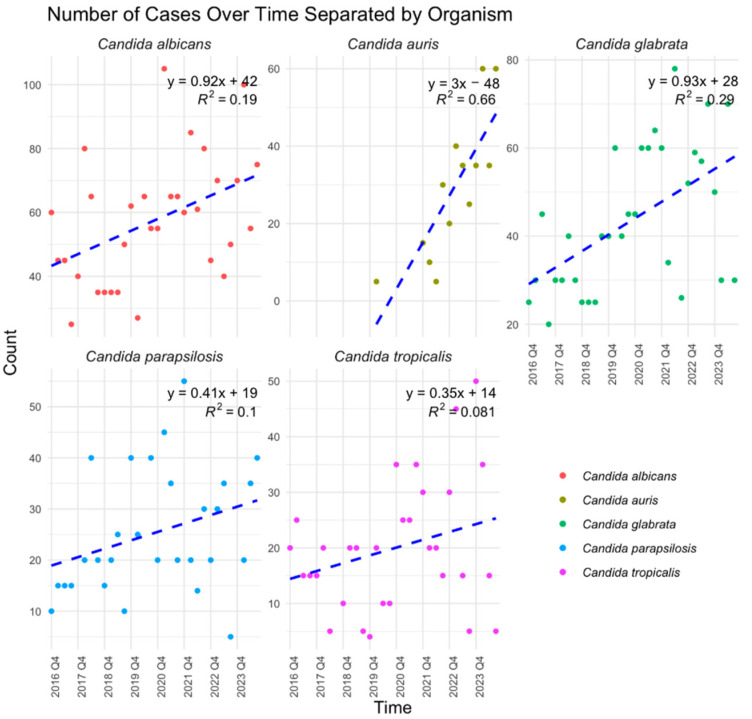
Linear regression models separated by each *Candida* species. There were significant increases in cases of *C. albicans* (*p* = 0.0121), *C. auris* (*p* = 0.0007), and *C. glabrata* (*p* = 0.0014) over the eight years of the study period. Q: quarter.

**Figure 3:**
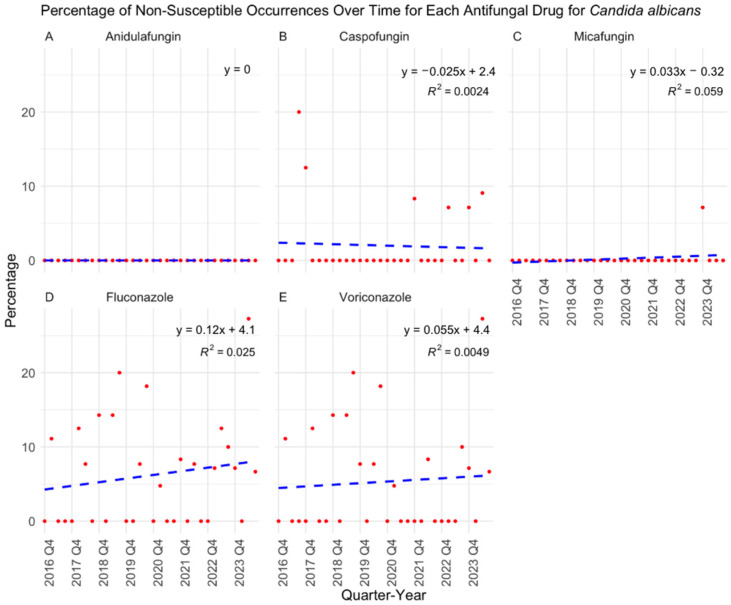
Percentage of non-susceptible occurrences over time for each antifungal drug in treating *Candida albicans* with results of a linear regression model. Throughout the eight-year study period, *C. albicans* maintained a consistent susceptibility profile, with sustained effectiveness against echinocandins (**A**–**C**), as indicated by low MICs. However, a gradual increase in fluconazole resistance was observed, though this trend did not reach statistical significance (*p* = 0.383) (**D**). Voriconazole remained largely effective, with only occasional isolates exhibiting slightly elevated MIC values (*p* = 0.704) (**E**). Q: quarter.

**Figure 4:**
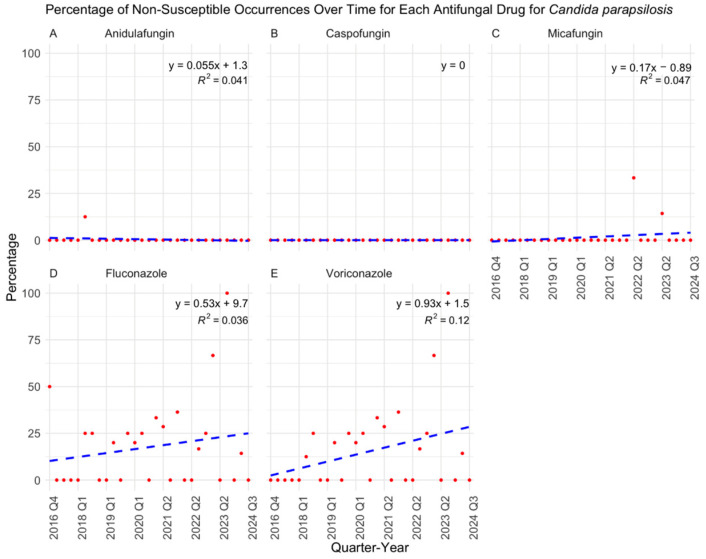
Percentage of non-susceptible occurrences over time for each antifungal drug in treating *Candida parapsilosis* with results of a linear regression model. *C. parapsilosis* remained largely susceptible to echinocandins (**A**–**C**). Although the increase in fluconazole and voriconazole resistances were not statistically significant (*p* = 0.346 and *p* = 0.074, respectively), a gradual upward trend was observed (**D**,**E**). Q: quarter.

**Figure 5:**
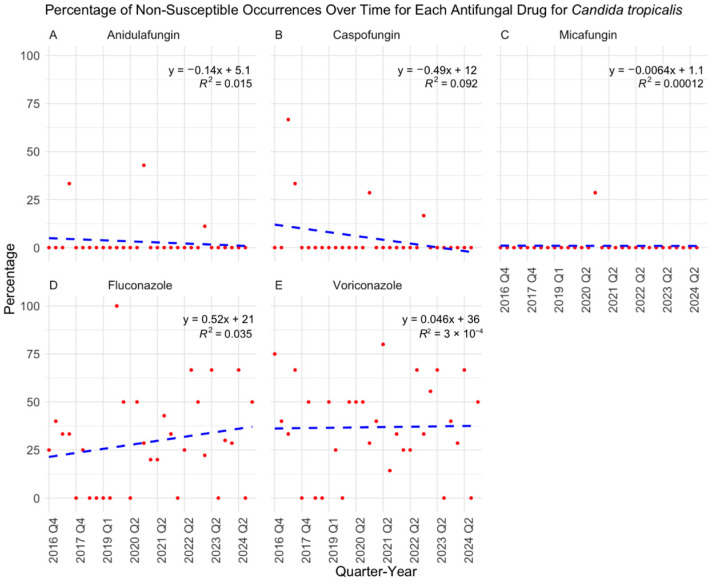
Percentage of non-susceptible occurrences over time for each antifungal drug in treating *Candida tropicalis* with results of a linear regression model. *C. tropicalis* remained largely susceptible to echinocandins with sporadic resistant isolates (**A**–**C**). More fluctuating susceptibility to fluconazole and voriconazole were observed with demonstrated elevated MIC values (**D**,**E**). Q: quarter.

**Figure 6:**
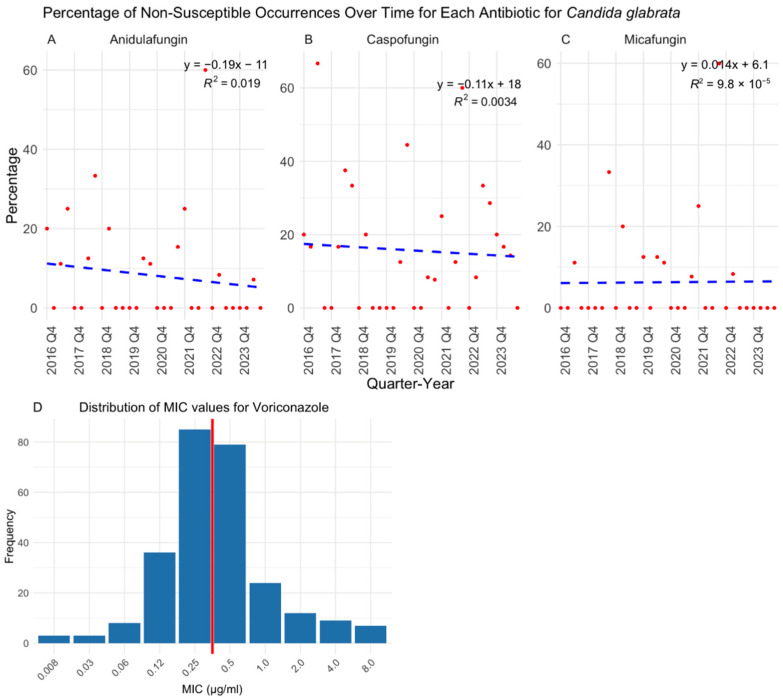
Percentage of non-susceptible occurrences over time for each antifungal drug in treating *Candida glabrata* with results of a linear regression model. Echinocandins remained largely effective against *C. glabrata*, though fluctuations in susceptibility were noted over time (**A**–**C**). 47.7% (135/283) of *C. glabrata* isolates had MIC values below epidemiological cutoff value (ECV, red solid line) of 0.25 µg/mL, while the majority exhibited elevated MICs (**D**). Q: quarter.

Over the eight-year study period, antifungal susceptibility testing volumes increased significantly (*p* < 0.0001) (Figure 1), driven primarily by the rising number of *C. albicans* (*p* = 0.0121), *C. auris* (*p* = 0.0007), and *C. glabrata* (*p* = 0.0014) isolates (Figure 2). While *C. albicans*, *C. parapsilosis*, and *C. tropicalis* remained consistently susceptible to echinocandins, fluconazole resistance showed a gradual but nonsignificant increase (Figure 3, Figure 4 and Figure 5). *C. glabrata* exhibited greater fluctuations in echinocandin susceptibility, with no clear trend observed (Figure 6). The distribution of fluconazole susceptible-dose dependent (SSD) vs. resistant *C. glabrata* isolates was approximately 6:1. According to CLSI guidelines, current data are insufficient to establish a correlation between in vitro susceptibility testing and clinical outcomes for *C. glabrata* and voriconazole. Slightly more than half of *C. glabrata* isolates in our population had MIC values exceeding the ECV of 0.25 µg/mL for voriconazole (Figure 6D).

**Figure 7:**
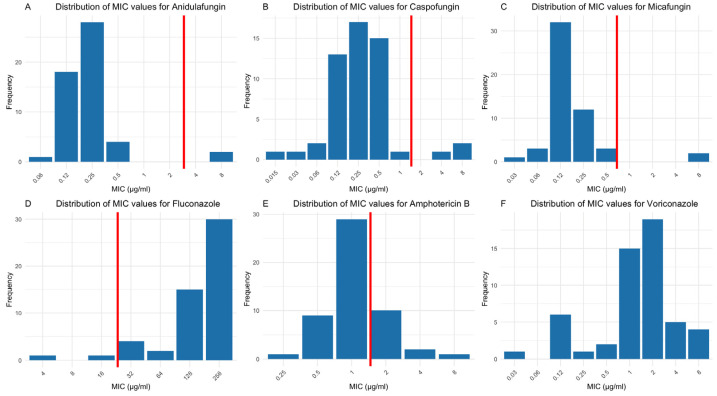
Distribution of MIC values for *Candida auris* when treated with various antifungals. *C. auris* demonstrated high MIC values against fluconazole (69/75 isolates, 92%, MIC ≥ 32 µg/mL) followed by amphotericin B (19/75 isolates, 32.2%, MIC ≥ 2 µg/mL) (**D**,**E**). Fewer isolates displayed elevated MICs for other antifungals (**A**–**C**). Red solid lines are the CDC’s tentative breakpoints (CDC-BP) for anidulafungin, caspofungin, fluconazole, and amphotericin B, as well as the ECV for micafungin. No CDC-BP or ECV for voriconazole is available (**F**).

Since there are no CLSI MIC interpretation guidelines for *C. auris* against various antifungal agents, the Centers for Disease Control and Prevention (CDC)’s tentative MIC breakpoints (CDC-BP) were used for fluconazole, anidulafungin, caspofungin, and amphotericin B (https://www.cdc.gov/candida-auris/hcp/laboratories/antifungal-susceptibility-testing.html accessed on 18 February 2025). The ECV was used for micafungin. Among *C. auris* isolates, 92% (69/75) had MIC values ≥ 32 µg/mL for fluconazole (Figure 7D), and 32.2% (19/75) had MIC values ≥ 2 µg/mL for amphotericin B (Figure 7E). Three isolates (4%) exceeded the CDC-BP for anidulafungin (MIC ≥ 4 µg/mL) (Figure 7A), while 4 isolates (5%) had MIC values above the CDC-BP for caspofungin (≥2 µg/mL) (Figure 7B). Additionally, 3 isolates (4%) had MIC values higher than the ECV (0.5 µg/mL) for micafungin (Figure 7C). There are no established CDC-BP or ECV for voriconazole; therefore, only the MIC distribution is illustrated (Figure 7F).

## 4. Discussion

This study showed a significant increase in antifungal susceptibility testing volumes over the 8-year study period, reflecting the growing clinical burden of *Candida* species. The potential rise in drug resistance emphasizes the need for continued regional surveillance to track resistance trends. While most *Candida* species remained susceptible to echinocandins, the increasing fluconazole resistance observed in *C. albicans*, *C. parapsilosis*, and *C. tropicalis*, as well as the newly emerged *C. auris* in our region aligns with CDC reports about 7% of all *Candida* blood samples being resistant to fluconazole [20]. Fluconazole resistance in *C. albicans* has also been reported in a university vaginitis referral clinic, with a rising trend observed over a 10-year collection period [21]. Additionally, since 2018, severe outbreaks caused by fluconazole-resistant *C. parapsilosis* strains have been documented worldwide [22,23]. Similarly, high rates of fluconazole-resistant *C. tropicalis* have been reported in both candiduria and invasive infections [24,25]. *C. glabrata* exhibited greater variability in susceptibility to both azoles and echinocandins, reinforcing the need for individualized susceptibility testing to guide optimal antifungal therapy.

The emergence of *C. auris* showed high resistance rates to fluconazole (92% of isolates with MIC ≥ 32) and amphotericin B (32.2% of isolates with MIC ≥ 2). Although these resistance rates were lower than those reported in the New York–New Jersy region [26], they still represent a major clinical concern. As a key driver of multidrug resistance, *C. auris* necessitates enhanced infection control measures, including environmental decontamination and contact precautions, as well as robust antifungal stewardship programs [27].

To address these challenges, future efforts should prioritize the development of novel antifungal agents and the integration of advanced diagnostic technologies, such as next-generation sequencing (NGS), to enable rapid identification and susceptibility profiling. Matrix-Assisted Laser Desorption/Ionization Time-of-Flight Mass Spectrometry (MALDI-TOF MS) and NGS-based approaches can significantly improve early detection and guide more effective treatment strategies [28,29]. Additionally, optimized antifungal use through a stewardship program is essential to mitigate resistance development, while empirical therapy should be guided by local susceptibility data. Future research should also explore adjunctive therapies, such as immunomodulators, to combat antifungal resistance. Expanding regional and global surveillance efforts will be vital for tracking emerging resistance patterns and informing policy decisions to combat the growing threat of multidrug-resistant *Candida* species.

This study has several limitations. First, as a retrospective analysis conducted at a single healthcare system in the Galveston–Houston Gulf Coast region, the findings may not be fully generalizable to other geographic areas. Second, the absence of MIC interpretation guidelines for *C. glabata* and *C. auris* with voriconazole required the use of ECV, which may not fully capture clinical resistance. Third, the study relied on data from the EPIC LIS, which may be subject to variation in testing practices over the eight-year period. Additionally, clinical outcomes and patient-specific factors were not assessed, limiting the ability to correlate resistance patterns with treatment efficacy. Despite these limitations, the study provides valuable insights into evolving antifungal resistance trends and highlights the need for continued surveillance and targeted intervention.

## 5. Conclusions

This eight-year retrospective study provides critical insights into evolving antifungal resistance patterns among *Candida* species in the Galveston–Houston Gulf Coast region. While *C. albicans*, *C. parapsilosis*, and *C. tropicalis* remained largely susceptible to echinocandins, increasing fluconazole resistance warrants continued surveillance. *C. glabrata* exhibited significant variability in susceptibility, reinforcing the need for individualized testing and treatment strategies. Most concerningly, *C. auris* emerged as a highly multidrug-resistant pathogen, with substantial resistance to fluconazole and amphotericin B.

These findings emphasize the urgent need for enhanced antifungal stewardship, improved diagnostics, and robust infection control measures. Expanding regional and global surveillance efforts and developing novel antifungal agents are crucial to addressing the challenges posed by multidrug-resistant fungi. An integrated approach combining surveillance, stewardship, and innovation is essential to mitigate the public health impact of antifungal resistance and improve patient outcomes.

## Data Availability

The original contributions presented in the study are included in the article. Further inquiries can be directed to the corresponding author.

## Supplementary Materials

The following supporting information can be downloaded at: https://www.mdpi.com/article/10.3390/jof11030232/s1, Table S1: List of yeasts with susceptibility data for the period of October 2016 to September 2024.
