# Supplementary material for: Trends in Antifungal Resistance Among Candida Species: An Eight-Year Retrospective Study in the Galveston–Houston Gulf Coast Region
Source: J Fungi (Basel). 2025 Mar 19;11(3):232. doi: 10.3390/jof11030232 (PMC11943608; doi:10.3390/jof11030232)
Supplement: Supplementary file 1 [file jof-11-00232-s001.zip › jof-3524835-supplementary.pdf]

Supplementary Table S1. List of yeasts with susceptibility data for the period of October 2016 to September 2024.

Yeast species	Number of isolates	Percentage (%)
Candida albicans	370	30.7
Candida auris	75	6.2
Candida bracarensis	2	0.2
Candida catenulata	1	0.1
Candida dubliniensis	40	3.3
Candida duobushaemulonii	5	0.4
Candida glabrata	283	23.5
Candida guilliermondii	7	0.6
Candida haemulonii	1	0.1
Kluyveromyces marxianus (Candida kefyr)	2	0.2
Pichia kudriavzevii (Candida krusei)	41	3.4
Yarrowia (Candida) lipolytica	1	0.1
Clavispora (Candida) lusitaniae	17	1.4
Pichia fermentans (Candida lambica)	3	0.2
Candida metapsilosis	5	0.4
Candida nivariensis	6	0.5
Candida orthopsilosis	8	0.7
Candida parapsilosis	147	12.2
Wickerhamomyces anomalus (Candida pelliculosa)	1	0.1
Candida tropicalis	125	10.4
Cryptococcus gattii (VGI)	1	0.1
Cryptococcus neoformans (VNI)	42	3.5
Kodamaea ohmeri	3	0.2
Rhodotorula minuta	2	0.2
Rhodotorula mucilaginosa	4	0.3
Saccharomyces cerevisiae	4	0.3
Scheffersomyces spartinae	1	0.1
Sporopachydermia lactativora	1	0.1
Trichosporon asahii	4	0.3
Yeast, unspecified	4	0.3
Total	1206	
